# Biodistribution of single and aggregated gold nanoparticles exposed to the human lung epithelial tissue barrier at the air-liquid interface

**DOI:** 10.1186/s12989-017-0231-3

**Published:** 2017-11-29

**Authors:** Estelle Durantie, Dimitri Vanhecke, Laura Rodriguez-Lorenzo, Flavien Delhaes, Sandor Balog, Dedy Septiadi, Joel Bourquin, Alke Petri-Fink, Barbara Rothen-Rutishauser

**Affiliations:** 1 BioNanomaterials Group, Adolphe Merkle Institute, Université de Fribourg, Chemin des Verdiers 4, 1700 Fribourg, Switzerland; 2 Chemistry Department, University of Fribourg, Chemin du Musée 9, 1700 Fribourg, Switzerland

**Keywords:** Aggregate, Gold nanoparticle, Air liquid interface cell exposure, Biodistribution, Human epithelial airway model, Translocation, Cellular uptake

## Abstract

**Background:**

The lung represents the primary entry route for airborne particles into the human body. Most studies addressed possible adverse effects using single (nano)particles, but aerosolic nanoparticles (NPs) tend to aggregate and form structures of several hundreds nm in diameter, changing the physico-chemical properties and interaction with cells. Our aim was to investigate how aggregation might affect the biodistribution; cellular uptake and translocation over time of aerosolized NPs at the air-blood barrier interface using a multicellular lung system.

**Results:**

Model gold nanoparticles (AuNPs) were engineered and well characterized to compare single NPs with aggregated NPs with hydrodynamic diameter of 32 and 106 nm, respectively. Exposures were performed by aerosolization of the particles onto the air-liquid interface of a three dimensional (3D) lung model. Particle deposition, cellular uptake and translocation kinetics of single and aggregated AuNPs were determined for various concentrations, (30, 60, 150 and 300 ng/cm^2^) and time points (4, 24 and 48 h) using transmission electron microscopy and inductively coupled plasma optical emission spectroscopy. No apparent harmful effect for single and aggregated AuNPs was observed by lactate dehydrogenase assay, nor pro-inflammation response by tumor necrosis factor α assessment. The cell layer integrity was also not impaired. The bio-distribution revealed that majority of the AuNPs, single or aggregated, were inside the cells, and only a minor fraction, less than 5%, was found on the basolateral side. No significant difference was observed in the translocation rate. However, aggregated AuNPs showed a significantly faster cellular uptake than single AuNPs at the first time point, i.e. 4 h.

**Conclusions:**

Our studies revealed that aggregated AuNPs showed significantly faster cellular uptake than single AuNPs at the first time point, i.e. 4 h, but the uptake rate was similar at later time points. In addition, aggregation did not affect translocation rate across the lung barrier model since similar translocation rates were observed for single as well as aggregated AuNPs.

**Electronic supplementary material:**

The online version of this article (10.1186/s12989-017-0231-3) contains supplementary material, which is available to authorized users.

## Background

Agglomeration and/or aggregation is an ubiquitous phenomenon observed for nanoparticles (NPs), however, the interaction of NP agglomerates with cells/tissues have only rarely being studied, consequently very little is known on their interaction with biological systems and subsequent fate [1–3]. Agglomerates and aggregates are secondary entities in which single NPs, or primary particles, are held together. In agglomerates, primary particles are assembled by weak physical interactions (i.e. van der Waals forces) and the whole process is reversible, while aggregates are defined as comprising strongly bonded primary particles, and the process is irreversible [4]. Agglomerates and aggregates will be simplified to the aggregates term from now on. These assembled NPs systems display more complex physicochemical properties than single NPs as their size, morphology, surface area and effective density will depend additionally on the fractal dimension and packing factors [5–7]. Combustion-derived NPs, are major contributors of aggregates in the airborne ambient air and, have been associated to adverse health effect [8, 9]. During the combustion process, i.e. diesel or gasoline engines, unburned or partially burned fuel undergo nucleation process forming single particles with diameter of about 10–30 nm [10, 11]. These single particles can further collapse to form aggregates with mean diameter below 100 nm up to several hundreds of nm which results in a reduced concentration number [6, 12, 13].

Humans are constantly exposed to airborne particles of different sources in the environment which enter the human body mainly by inhalation. NPs with a diameter from 5 to 500 nm can enter and penetrate into the alveolar region of the lung by diffusion processed [14] and the deposited NPs have been shown to translocate across the air-blood barrier reaching the blood or lymphatic circulation, to be further distributed to secondary organs [15–19]. Evidence suggest that NPs’ translocation in healthy lungs most likely occurs via transcellular rather than paracellular pathway [20]. Moreover, findings support active processes, e.g. endocytotic uptake mechanism, to be preferentially involved, albeit passive diffusion is not excluded. It has been shown that NPs’ physicochemical properties, such as size, shape and surface, influence their uptake into cells and transport across the lung barrier (translocation) [21]. Some studies in rats have reported a higher translocation rate of smaller NPs [17, 19, 22, 23], while in others studies NP surface charge was found to influence the translocation [16]. Although aggregation is a common phenomenon, most of the in vitro and in vivo studies assume NPs remain in a single state when studying interactions with cellular, tissue or organ structures. There are only few investigations explicitly on the interactions of aggregates with biological systems (for a review see ref. [24]). Among the very rare in vivo studies, the effect of the aggregate size or effect of the primary particle size were explored in rats. It has been shown that smaller aggregates, 20 vs 80 nm, containing same primary single iridium NPs (2.4 nm) [19] or smaller primary particle size AuNPs, 7 vs 20 nm, forming aggregates with peak diameter of 45 nm [25] exhibited higher translocation and wider distribution to secondary organs. However, systematic in-depth studies at the mechanistic level comparing single particles with aggregates are still missing.

In the present work, the behavior of single and aggregated AuNPs was compared regarding their biodistribution across the air-blood tissue barrier by investigating their cellular uptake and translocation at different time points (i.e. 4, 24 and 48 h). Well-defined single and aggregated particles were used as model particles with hydrodynamic diameters of 32 and 106 nm, respectively, composed of primary AuNPs of 14.5 nm (core diameter) stabilized with polymer mixture consisting of polyvinyl alcohol and polyallyl amine (PVA/PAAm). To simulate a realistic inhalation the NPs were deposited at the air-liquid interface onto an in vitro 3D human alveolar epithelial barrier. The 3D human lung model, developed by Rothen-Rutishauser et al. [26], is composed of human lung alveolar cells (A549 cell line), primary human-monocyte derived macrophages and dendritic cells. The cells were exposed to single and aggregated AuNPs at the air-liquid interface at four different concentrations, i.e. 30, 60, 150 and 300 ng/cm^2^, and the deposition was thoroughly characterized by inductively coupled plasma optical emission spectrometry (ICP-OES) and transmission electron microscopy (TEM). Cytotoxicity, pro-inflammation and cell layer integrity were assessed at 4, 24 and 48 h after exposure. NPs cellular uptake and translocation were then assessed by measuring the mass of gold by ICP-OES in the individual compartments (i.e. apical side, inside the tissue and in the basal medium). In addition, localization of intracellular NPs was analyzed by TEM.

## Methods

### AuNP synthesis

Single and aggregated AuNPs were prepared following the procedure of Hirsch et al. with an adaptation of the polymer coating [27].

Synthesis of tiopronin-coated AuNPs: Briefly, to a solution of tetrachloroauric acid (500 mL, 0.5 mm; Sigma Aldrich Chemie GmbH, Buchs, Switzerland) in ultrapure water (MilliQ H_2_O, Merck Millipore) heated at reflux was added quickly a warmed solution of sodium citrate (25 mL, 1% *w*/*v*) and stirred for 15 min. The reaction mixture was cooled to room temperature and a solution of tiopronin (2-mercaptopropionylglycine; Sigma-Aldrich) (15.5 mL, 0.5 mm) was added. The reaction mixture was stirred at room temperature overnight.

Preparation of polymer mixture PVA/PAAm-(17 kDa) and PVA/PAAm-(65 kDa): The mixtures of poly(vinyl alcohol) (PVA) and poly(allylamine) (PAAm), PVA/PAAm-(17 kDa) and PVA/PAAm-(65 kDa) were prepared by dissolving PVA (11.8% *w*/*v*; 5.9 g; 14 kDa, Mowiol 3–85, Omya AG, Switzerland) and PAAm 17 kDa (0.2% w/v; 490 μL; Fluka solution 20% wt) or PAAm 65 kDa (0.2% w/v; 980 μL; Fluka solution 10% wt), respectively, in MilliQ H_2_O (final volume 50 mL) and stirred overnight.

In the polymer mixtures PVA/PAAm (11.8:0.2, mass ratio), the PAAm size was changed depending if it was used to coat single or aggregated AuNPs. The PAAm 65 kDa was chosen to ensure the electrosteric stabilization of the assembly while PAAm 17 kDa was used to coat single AuNPs, hence avoiding any aggregation due to polymer length. However, to make comparative study of the two systems, it is important to note that amount of amines remains equal in the two mixtures.

Preparation of single AuNPs: The solution of tiopronin-coated AuNPs (100 mL) was added dropwise to the aqueous polymer mixture PVA/PAAm-(17 kDa) (9 mL) and stirred for 4 h at room temperature. After 1 day of storage at 4 °C, the suspension was centrifuged at 10000 × *g* for 1 h and the supernatant was collected and centrifuged again under the same conditions. This process was repeated one more time.

Preparation of aggregates AuNPs: The solution of tiopronin-coated AuNPs (10 mL) was treated with HCl (1 m, 36 μL) so that the mixture reaches a pH of 3. An aqueous mixture of polymer PVA/PAAm-(65 kDa) was added to stabilize the agglomerates. After 1 day of storage at 4 °C, the suspension was centrifuged at 5000 × *g* for 1 h. This process was repeated one more time.

### AuNPs characterisation

#### UV-Vis spectroscopy

UV-Vis spectra of the single and aggregated AuNPs were recorded in MilliQ H_2_O using a Jasco V-670 spectrophotometer (Jasco Europe S.R.L., Milano, Italy) with 10 mm optical pathlength optical glass cuvettes. Concentration of AuNPs suspensions were determined by the absorbance intensity at 400 nm as described by Scarabelli et al. [28].

#### Transmission electron microscopy (TEM)

All samples were measured with an FEI Tecnai spirit TEM (FEI, Hillsboro, Oregon, USA) at 120 kV. Images were recorded with a Veleta CCD camera 2048 × 2048 (Olympus-SIS, Münster, Germany) or Eagle CCD camera 4096 × 4096 (FEI, Hillsboro, Oregon, USA) and processed using ImageJ software as described in the supporting information (Section 1 of Additional file 1: Supplementary information).

Single AuNPs were characterized using conventional TEM: Briefly, single AuNPs suspension (10 μL) was deposited onto a 400 mesh carbon-coated copper grid and let dried at room temperature. Images were recorded with the Veleta camera.

Aggregated AuNPs were characterized using cryo-TEM: Aggregated AuNPs suspension (5 μL) was deposited on a carbon-coated copper grid and liquid excess was carefully removed with filter paper. The grid was then plunged into a liquid ethane bath cooled by liquid nitrogen. The resulting vitrified sample was then stored in liquid nitrogen prior to analysis. Images were recorded using the Eagle camera.

#### Depolarized dynamic light scattering (DDLS)

Light scattering data were collected at constant temperature (21 °C) at θ = 15°, using a commercial goniometer instrument (3D LS Spectrometer, LS Instruments AG, Switzerland). The primary beam was formed by a linearly polarized and collimated laser beam (Cobalt 05–01 diode pumped solid state laser, λ = 660 nm, P max. = 500 mW), and the scattered light was collected by single-mode optical fibres equipped with integrated collimation optics. The collected light was coupled into two high-sensitivity APD detectors via laser-line filters (Perkin Elmer, Single Photon Counting Module), and their outputs were fed into a two-channel multiple-tau correlator. The signal-to-noise ratio was improved by cross-correlating these two channels. With respect to the primary beam, depolarized scattering was observed via cross-polarizers. The incoming laser beam passed through a Glan-Thompson polarizer with an extinction ratio of 10^−6^, and another Glan-Thompson polarizer, with an extinction ratio of 10^−8^, was mounted in front of the collection optics. To estimate the number-averaged particle size distribution, the DDLS spectra were analyzed by the approach presented elsewhere [29].

### 3D human epithelial tissue barrier model

#### Cell culture

Experiments were carried out using the human alveolar epithelial type II cell line A549 [30], human blood monocyte-derived macrophages (MDM) and dendritic cells (MDDC). A549 cell line was obtained from the American Type Culture Collection (ATCC, USA), while human whole blood monocytes (MDM and MDDC) were isolated from buffy coats provided by the blood donation service SRK Bern and purified using CD14 Microbeads (Milteny Biotech) [31]. Cells were maintained in RPMI 1640 (Gibco, Life Technologies Europe B.V., Zug, Switzerland) supplemented with 10% (*v*/v) fetal bovine serum (FBS; PAA Laboratories, Chemie Brunschwig AG, Basel, Switzerland), 1% (v/v) L-Glutamine (Life Technologies Europe) and 1% (v/v) penicillin/streptomycin (Gibco) and placed in a humidified incubator (37 °C, 5% CO_2_). A549 cells were subcultured through trypsinization when reached near-confluence and medium was changed every 3 days. Initial cell concentrations were calculated using trypan blue exclusion method (0.4% Trypan blue solution, T8154; Sigma Aldrich). The working cell concentrations were prepared by diluting cells with cell culture medium.

#### 3D co-culture model

The co-cultures were prepared as previously described [26]. Briefly, A549 cells (54⋅10^4^ cells/mL, 0.5 mL, upper chamber) were seeded on a transparent BD Falcon cell culture inserts (surface area of 0.9 cm^2^, pores of 3.0 μm diameter, PET membranes for 12-well plates; BD Biosciences) placed in a BD Falcon tissue culture plates (12-er well plates; BD Biosciences) containing 1.5 mL medium (lower chamber). Cells were cultured for 4 days and the medium was changed after the 2nd day. On day 5, medium was removed from the upper and lower chambers, the inserts were turned up-side down, placed in a petri dish and cells at the bottom of the membrane were gently abraded with a cell scraper. MDDCs (84⋅10^4^ cells/mL, 65 μL) were then pipetted onto the bottom side of the inserts and incubated for 70 min. Afterwards, the insert were placed back into the well plate containing 1.5 mL fresh medium. Finally MDMs (2.5⋅10^4^ cells/mL, 0.5 mL) were added on top of the A549 cells prior to be incubated for another 24 h. The cells were then transferred from submerged to air-liquid interface conditions 24 h prior to be exposed. On day 6, medium was removed from the upper chamber and medium form the lower chamber was replaced with 0.6 mL of fresh medium.

#### Characterization of co-culture model with laser scanning microscopy (LSM)

LSM description: Samples were acquired using LSM 710 Meta with an inverted Zeiss microscope (Carl Zeiss GmbH, Jena, Germany). The z-stack images of the cells were acquired using 20× and 40× magnification lens with numerical aperture with numerical aperture, NA 0.75 and 1.3, respectively. Image processing was performed using ImageJ software and 3D rendering with Imaris software.

Visualization of the 3D co-culture model: In order to obtain a clear characterization of the 3D co-culture model, each cell type was stained with different fluorophores (Vybrant® multicolor cell labeling kit, Invitrogen Molecular Probes) prior to the co-culture composition. Briefly, MDDCs and MDMs (1.10^6^ cells/mL in RPMI 1640) were treated with vibrant dye DiI and DiD, respectively, (5 μL for 1 mL of cell suspension) and incubated for 20 min in the incubator. Cells were centrifuged and washed with RPMI 1640 3 times prior to be seeded. In the meantime, the layer of A549 grown on the insert was treated with vibrant dye DiO (1.5 μL in 200 μL of RPMI 1640) and incubated for 20 min in the incubator. Cells were washed twice with RPMI 1640 for 10 min. The co-cultures were then composed as previously described except that MDMs were seeded at a higher density (4⋅10^4^ cells/mL) in order to improve their visualization in the window frame.

### Air-liquid Interface cell exposure

#### Exposure system

The cells were exposed to AuNPs at the air-liquid interface using the Vitrocell® Cloud exposure system. It consists of three main parts: a nebulizer, an aerosol chamber and a base module constituted of 12-well size inserts and connected to a controlled heating unit. The aerosol is generated into the exposure chamber via a vibrating mesh (Aeroneb®Pro, Aerogen, with a span of 2.5–6.0 μm volume mean diameter). The Vitrocell® Cloud exposure system allows for a dose-controlled and uniform deposition.

#### Cell exposure

Cells were exposed at the air-liquid interface to single and aggregated AuNPs by nebulization of 200 μL of AuNPs suspension in 0.5 mm NaCl_aq_ at the specific concentrations of 0.05, 0.10, 0.25 and 0.50 mg/mL (concentrations determined by UV-Vis measurements). Exposure to 200 μL of 0.5 mm NaCl_aq_ only was done as negative control. After 10 min exposure, deposition of cloud was complete and the TCCC were kept at the air-liquid interface in fresh medium for post-exposure incubation times of 4, 24 and 48 h in the incubator.

### Deposition characterization

The deposition of AuNPs after nebulization was analyzed by inductively coupled plasma optical emission spectroscopy (ICP-OES) and transmission electron microscopy (TEM) to determine the mass of gold and the number of particles (i.e. single or aggregates), respectively.

#### TEM analysis

For each exposure condition (single/aggregated AuNP and concentration), a single slot copper grid was exposed to nebulized AuNPs. Deposition was repeated in triplicate for each condition. Automatic acquisition of 25 images per grid was recorded using the Eagle camera at a magnification of 18,500X (image size 2.39 × 2.39 μm). Images containing obvious large artefacts (i.e. dirt, grid edge) that interfered with the automated thresholding were excluded prior to the image processing, since these data yield biased results. Also empty pictures were not processed. Image processing was performed on a stack of images as described in the supporting information (Section 1 of Additional file 1: Supplementary information) and the number of analyzed particles is also reported. The output of the analysis is the number of events (single particles or aggregates) and their associated area. Hence, the number deposition (single and aggregated AuNPs/cm^2^) and surface deposition (% area) can be obtained by the ratio of these results and the total surface area of all pictures (including empty pictures).

#### ICP-OES analysis

Mass deposition was determined by addition of the mass of each fraction (apical, intracellular and basal). See chapter below.

### Biological response

Cell morphology, cell layer integrity, cytotoxicity and (*pro*)-inflammation of co-culture model exposed to AuNPs were evaluated and compared to co-culture model exposed to 0.5 mm aqueous NaCl as negative control. Assays were repeated in 3 individual experiments.

#### Cell morphology after exposure was analyzed by LSM (see LSM description above)

After particle exposure and post-incubation, the samples were washed with PBS, fixed with paraformaldehyde (4% in PBS, Sigma Aldrich) for 10 to 15 min and washed twice with PBS. The samples were incubated with DAPI (4′,6-diamidino-2-phenylindole, Sigma Aldrich) (1:100, nuclei stain) and rhodamine phalloidin (1:50, F-actin cytoskeleton stain, Life Technologies) in Triton X-100 solution (0.2% in PBS, to permeabilize the cell membrane, Sigma Aldrich) for 60 min. Afterwards, the samples were washed with PBS (3 times) prior to be mounted on objective slides in glycergel mounting medium (Dako).

#### Cell layer integrity

Permeability to fluorescein isothiocyanate (FITC) coupled to dextran 70 kDa (Sigma Aldrich) was used to assess cell layer integrity. Intact epithelial cell layer grown on a membrane forming tight junction should prevent paracellular transport, and so transport of FITC dextran applied on the apical side is low. Briefly, after post-exposure time, TCCC was rinsed with RPMI without phenol red and the medium in the basal side was replaced by 1.5 mL of RPMI without phenol red. At the apical side, 500 μL of 1 mg/mL FITC dextran in RPMI without phenol red was applied and incubated for 1 h. As a positive control, EDTA (Ethylenediaminetetraacetic Acid, Sigma Aldrich) 10 mM in RPMI without phenol red was added to the FITC dextran solution applied at the apical side. Afterwards, medium in the lower side was collected and the passage of FITC dextran was quantified by measuring fluorescence of the sample in triplicate using a Multireader microplate (λ_ex/λem_ = 490/520 nm). The fluorescence was normalized to the translocation of the FITC dextran trough an empty insert.

#### Cytotoxicity

The cytotoxicity was assessed by measuring the release of the cytosolic enzyme lactate dehydrogenase (LDH) into the medium (basal compartment) that is indicative of cell membrane damage. LDH was quantified using the LDH cytotoxicity detection kit (Roche Applied Science, Germany) according to the manufacturer’s guidelines and absorbance was read at 490 nm using a microplate reader (Bio-Rad). The sample was measured in triplicate, sample absorbance was corrected by subtracting medium absorbance and values were then normalized to the negative control. As a positive control, TCCC were treated with 100 μL of 0.2% triton X100 in PBS at the apical side and placed in the incubator for the post-exposure time. Positive control sample was diluted 10 times prior to be measured to remain in the linear part of the LDH activity and the absorbance was then multiply by the diluting factor to express the normalized LDH activity.

#### (pro)-inflammation

The (pro)-inflammation response was investigated by quantifying the tumor necrosis factor α (TNF-α) release into the medium (basal compartment) using the DuoSet ELISA Development Kit (R&D Systems) according to the manufacturer’s protocol and absorbance was read at 450 nm using a microplate reader (Bio-Rad). The sample was measured in triplicates, sample absorbance was corrected by subtracting medium absorbance. As a positive control, TCCC were treated with 600 μL of LPS 1 μg/mL in medium at the basal side and placed in the incubator for the post-exposure time.

### Particle distribution

For each exposure condition (i.e. AuNPs type, concentration and time), apical, intracellular and basal fractions were collected from 3 different inserts and the amount of gold was determined using ICP-OES. Each exposure condition was repeated in 4 individual experiments.

#### Sample collection

First, the basolateral medium was collected. Then, to recover particles deposited on the apical side that have not been associated with cells, the cell layer was washed twice with PBS (300 μL). Finally, the cells were collected by scrapping the cells after treatment with trypsin-EDTA (600 μL) for approximatively 25 min at 37 °C. The obtained samples were stored at −80 °C until further processing.

#### Sample preparation

Microwave-assisted acidic digestion of AuNP samples was conducted in the microwave Multiwave Pro (Anton Paar, Germany) which is equipped with two standard magnetrons of 850 W able to deliver a microwave power up to 1500 W in an unpulsed mode over the full power range. The applied microwave energy is controlled by contactless sensors for internal temperature and vessel pressure and by IR sensor which is equipped with a temperature sensor, preventing overheating, and an IR sensor monitors the temperature of vessels. Rotor 24HVT50 was used with pressure vessels HVT50 made of PTFE-TFM. Briefly, samples were gradually defrosted from −80 °C → -20 °C → 4 °C → room temperature. Samples were transferred into vessels and were treated with HNO_3_ 70% (0.6 mL, Sigma Adrich) and H_2_O_2_ 30% (0.3 mL, Merck). The volume was completed to 3 mL with millipore H_2_O (0.3 mL). Samples were placed in the microwave and the mixture was irradiated at 100 °C (ramp for 10 min, hold 10 min) and further at 140 °C (ramp for 10 min, hold for 10 min) and finally cooled to 70 °C (ramp 12 min) with a maximum power of 600 W. Samples was then transferred into 15 mL falcon tubes, and each vessel was rinsed with millipore H_2_O (0.5 mL). Finally samples were completed with millipore H_2_O, if necessary, to a final volume of 3.5 mL.

#### ICP-OES

ICP-OES analyses were carried out on an Optima 7000 DV, Perkin Elmer. Measurements were performed at a wavelength of 242.8 nm, at an axial plasma view. The plasma flow was 15 L/min and the sample flow rate 1.5 mL/min. Calibration was performed using a gold standard for ICP (1001 mg/L ± 2 mg/L, Fluka) for each matrix from 0.025 to 0.5 μg/mL. Samples were measured in triplicate and a washing step was performed by the instrument between each exposure condition. Analysis was repeated 4 times for each exposure condition. The obtained concentration (μg/mL) was multiplied by the sample volume (3.5 mL) to determine the Au mass.

### Particle localization in the co-culture by TEM

Sample preparation: All chemicals were obtained from Polysciences, unless otherwise stated. The exposed cells on the transwell membrane were fixed with glutaraldehyde 2.5% in HEPES buffer (0.03 m) for at least 48 h at 4 °C. Then, they were washed three times at room temperature with sodium cacodylate buffer (0.1 m) under gentle stirring for at least 5 min per washing step. The samples were then post-fixed with OsO_4_ (1% in 0.1 m sodium cacodylate buffer) for 2 h and washed again three times with sodium cacodylate buffer as described before. The dehydration of the samples was achieved using a series of gradually increasing ethanol concentration (30, 50, 70, 80, 96 and 100% dried over molecular sieve), each step lasting 15 min followed by gradual increasing concentrations of the epoxy embedding polymer in 100% ethanol dried over molecular sieve (30% for 1 h at 4 °C, 70% overnight at 4 °C, 100% for 2 h at room temperature, repeated twice). The samples were then polymerized at 60 °C for 72 h. From the polymerized epoxy resin blocks, ultrathin sections of 80 nm (gray-sliver reflection) were sectioned perpendicular to the Transwell membrane by a Ultra 35° diamond knife (Diatome, Nidau, Switzerland) using a Leica Ultracut UC6 ultramicrotome (Leica Microsystems, Wetzlar, Germany). These sections were then brought onto Formvar film coated single slot copper grids (PlanoEM, Wetzlar, Germany) and stained with uranyl acetate and lead citrate in a Leica EM Stain citrate (Leica Microsystems, Wetzlar, Germany). TEM images were recorded using the Veleta camera, with a resolution of 11.2 nm/pixel (overviews) and 0.78 nm/pixel (details).

### Data analysis

Number of repetitions is given in the respective paragraph. Individual experiments mean different aerosolization and cell culture with different passage number and different monocytes isolations. Data are presented either as mean ± standard deviation or mean with data points. Statistics were run using ORIGINLAB. Statistical differences were determined by comparison of the means using one-way ANOVA and Tukey’s honest significance difference test (*p* = 0.05).

## Results

### Particle AuNPs synthesis and characterization

**Fig. 1:**
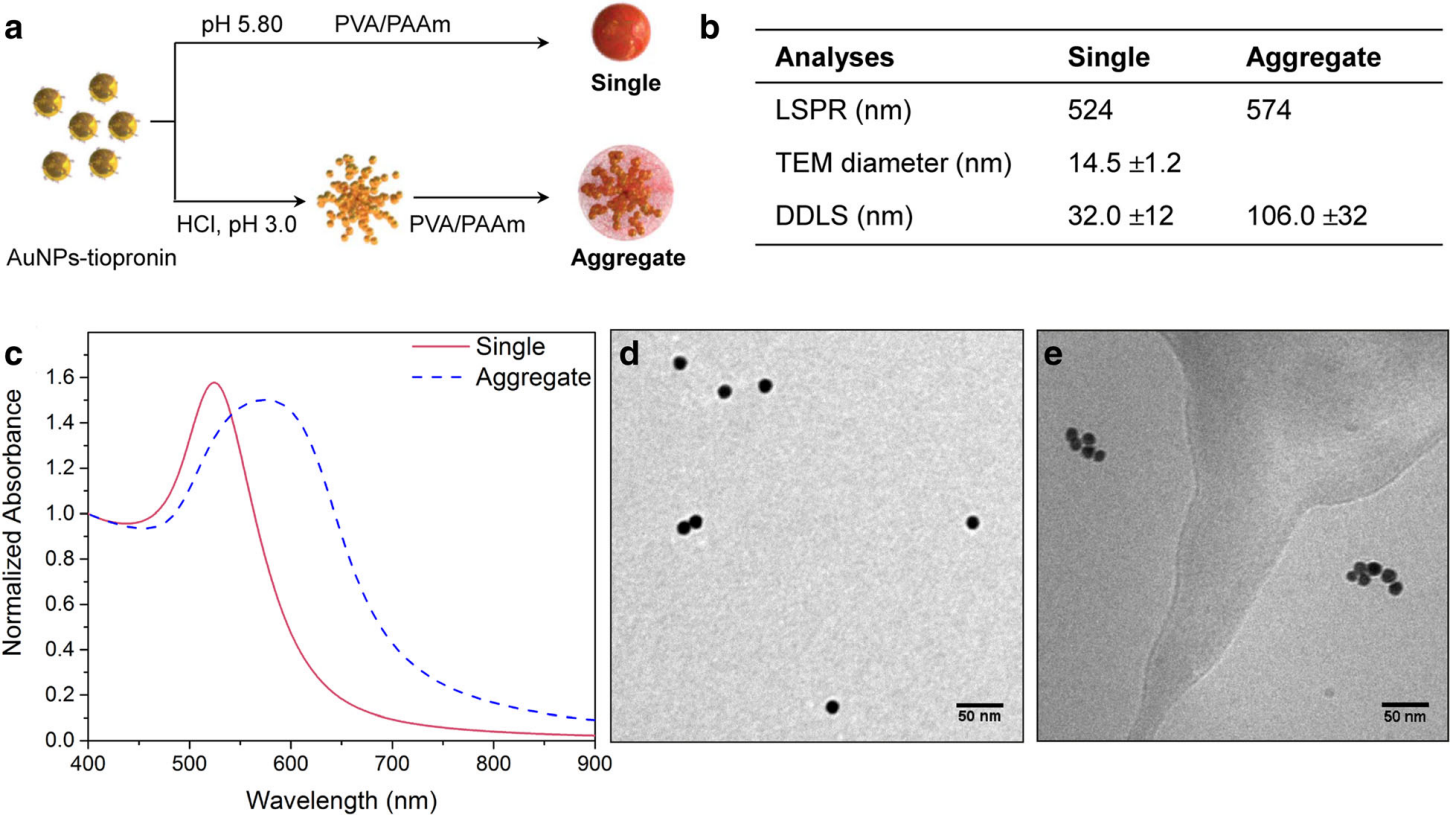
Characterisation of single and aggregated AuNPs. **a** Preparation of single and aggregated AuNPs from AuNPs-tiopronin. Scheme modified from Hirsh et al. with permission [27]. **b** Physicochemical parameters of single and aggregated AuNPs. **c** Extinction spectra of single and aggregated AuNPs. The spectra were normalized based on the absorbance at 400 nm. **d** TEM image of single AuNPs. **e** cryo-TEM image of aggregated AuNPs

Single and aggregated AuNPs were prepared adapting the method developed by Hirsch et al. (Fig. 1a) [27]. Citrate-capped AuNPs were covalently functionalized with tiopronin forming AuNPs which then displayed carboxylic groups on the surface. As shown in the previous study, protonation of these carboxylic groups allows the formation of controlled aggregates. The agglomeration process is driven by electrostatic attraction between two tiopronin-AuNPs resulting from the hydrogen bonds formation, hence replacing the electrostatic repulsion when carboxylic groups are negatively charged. Agglomeration was then stopped by addition of a polymer mixture composed of polyvinyl alcohol and polyallyl amine (PVA/PAAm). PVA/PAAm is important for stabilizing the agglomerates to aggregates, because (i) amine groups act as proton sponge, (ii) they interact with the negative charges of the tiopronin-AuNPs and (iii) the overall PVA/PAAm coating prevents the reversal of the self-assembly at physiological pH. Single tiopronin-AuNPs were also coated with PVA/PAAm polymers to have similar chemical surface properties.

The synthesized single and aggregated AuNPs were then characterized by UV-Vis spectroscopy, TEM, DDLS and zeta-potential, the results are summarized in the table (Fig. 1b). UV-Vis spectra (Fig. 1c) showed a red shift from 524 to 574 nm and a broadened localized surface plasmon resonance (LSPR) band for the aggregates in comparison to the single NPs showing the formation of the aggregates. TEM images and DDLS measurements further confirmed the size and state of dispersion of the different samples. Single AuNPs showed a core size of 14.5 nm (TEM) (Fig. 1d, Additional file 1: Figure S5) and a hydrodynamic diameter of 32 ± 12 nm (DDLS). The difference of size given by TEM and DDLS was due to the large PVA polymer coating. TEM images of aggregated AuNPs were taken at cryogenic temperature to prevent aggregates resulting from drying artefacts (Fig. 1e, Additional file 1: Figure S5). TEM images analysis showed aggregates with heterogeneous population and a mean of 4 single AuNPs per aggregate (Additional file 1: Figure S6). DDLS measurements confirmed also the aggregates formation giving a hydrodynamic diameter of 106 ± 32 nm. Finally, the near neutral or positive zeta potential of single and aggregated AuNPs indicates the presence of the PVA/PAAm coating (Additional file 1: Table S2).

### AuNP aerosolization and characterization of deposition

Deposition of the aerosolized single and aggregated AuNPs was thoroughly analyzed using ICP-OES and TEM techniques.

**Fig. 2:**
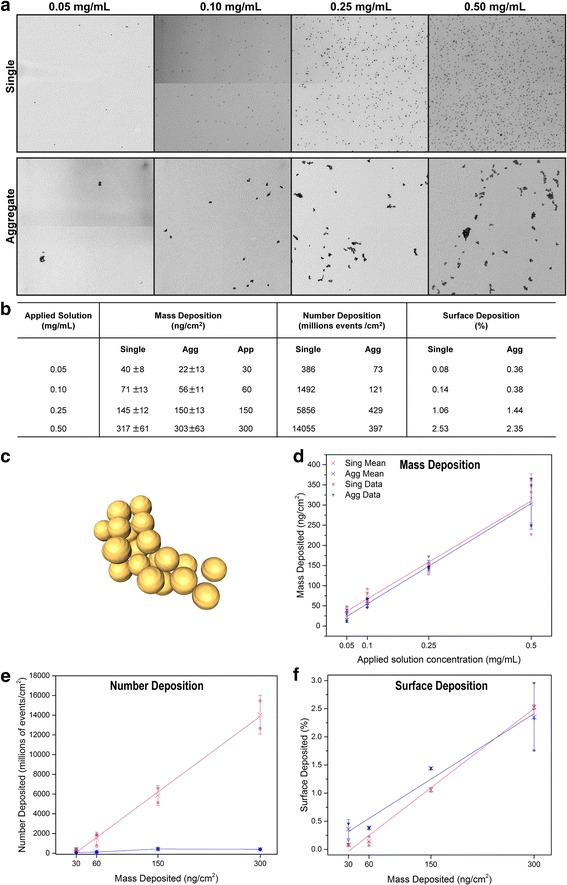
Deposition characterization. **a** Deposition pictures of each concentration for single and aggregated AuNPs. **b** Characterization of the deposition. The mass obtained after single and aggregated AuNPs deposition was statistically not different and were simplified to an approximate (App) mass for further discussions. **c** 3D reconstruction of a deposited aggregated AuNP by TEM tomography [47]. **d** Mass deposition of single and aggregated AuNPs. **e** Number of events deposition of single and aggregated AuNPs. **f** Surface deposition of single and aggregated AuNPs. Legend graph **d**, **e**, **f**: cross (x) represents mean, whisker: standard deviation

TEM images obtained from the deposited single and aggregated AuNPs showed that the particle state (i.e. single and aggregate) was generally not affected by aerosolization and deposition processes (Fig. 2a). The particle diameter remained similar to prior aerosolization.

Deposition was quantified on a specific surface area by determining (i) the mass of deposited gold using ICP-OES, (ii) the number and (iii) the surface of deposited single or aggregated AuNPs using TEM, resulting in the mass deposition (ng/cm^2^), the number deposition (# events/cm^2^) and the surface deposition (%), respectively. The results are summarized in the table as mean values for each aerosolized concentration of single and aggregated AuNPs (Fig. 2b). TEM images analysis processing is explained in the supplementary information (Section 1 of Additional file 1: Supplementary information) and a 3D reconstruction of deposited aggregated is shown in Fig. 2c. Analysis of the mass deposition expressed in function of the aerosolized dose (mg/mL), showed that the deposition was reproducible and proportional to the applied dose for both AuNPs systems (Fig. 2d, linear fit r^2^ > 0.99). It is important to note that the deposited mass obtained after aerosolization of single and aggregated AuNPs were not statistically different and were simplified to an approximate mass, from 30 to 300 ng/cm^2^, for further discussions (Fig. 2b). On the other hand, deposition quantification counting the number of events, showed an important difference between single and aggregated AuNPs, especially at the highest doses (Fig. 2e). Numbers of single AuNPs were 10 to 30 fold more than the aggregated ones at the deposited mass of 150 and 300 ng/cm^2^, respectively. Interestingly, while the number of deposited single AuNPs increased proportionally to the deposited mass, the number of deposited aggregates did not increase at doses above 150 ng/cm^2^. Indeed, visualization of the TEM images showed that at the highest concentrations (Fig. 2a, 0.50 mg/mL), aggregates tended to form bigger clusters while single particles remained mainly dispersed. Finally, analysis of the surface deposition showed that the deposited single and aggregated NPs covered similar total surface area which increased proportionally with the deposited mass (Fig. 2f). This is also supporting the previous observation of a clustering effect with the aggregates at higher concentration. Indeed, to obtain the same total surface area as the single NPs, the individual deposited aggregated NPs must have larger surface area to compensate the lower number deposition. Determination of the number of particles per aggregates showed an increase at the highest concentration (300 ng/cm^2^) (Additional file 1: Figure S6). The larger size of the deposited aggregates at higher concentration could be the result of agglomeration occurring during nebulization, as also observed for higher concentration of nebulized superparamagnetic iron oxide NPs [32] and/or the result of the drying artifact of the more concentrated nebulized droplets.

### Characterization of the 3D human epithelial tissue barrier after AuNPs exposure

**Fig. 3:**
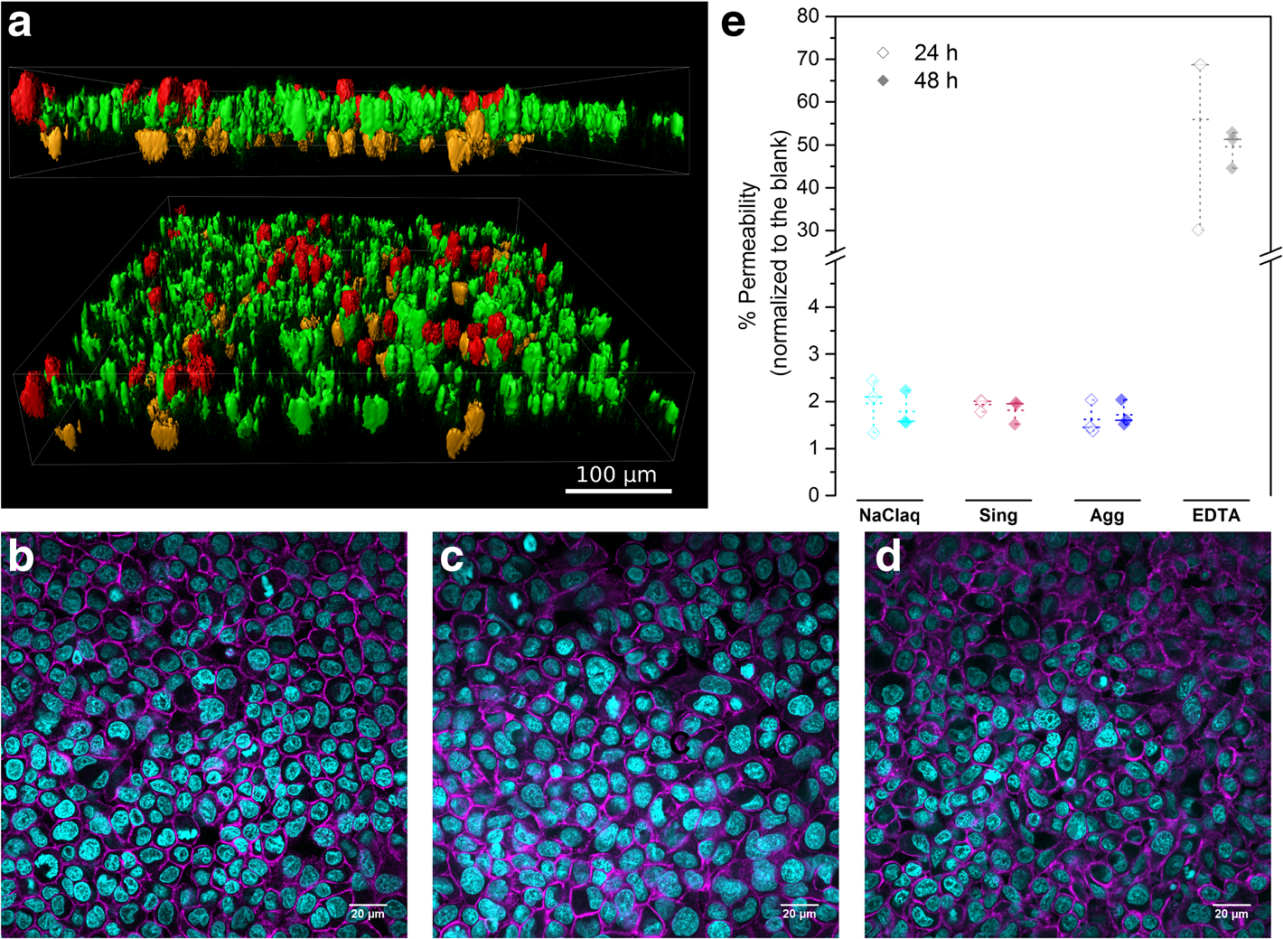
Characterization of 3D human epithelial tissue barrier. **a** Laser Scanning Microscopy (LSM) of 3D co-culture model after 3D rendering: epithelial cells (green), macrophages (red), and dendritic cells (orange). The triple layer co-culture with macrophages on top and dendritic cells at the bottom is shown from two different views. **b**-**d** LSM images of F-actin (magenta) and nuclei (cyan) 24 h after exposure to **b**. NaCl_aq_ solution, **c** Single AuNPs 60 ng/cm^2^, **d** Aggregated AuNPs 60 ng/cm^2^. **e** Translocation of FITC-Dextran 70 kDa accross the co-culture 24 and 48 h after-exposure to single and aggregated AuNPs (300 ng/cm^2^). The fluorescence in the baso-lateral compartment was measured and normalized to the blank control (empty insert, without cells). Sing = single; Agg = aggregate. Plain horizontal line represents the mean value and dashed horizontal line the median

The in vitro lung model was first characterized by LSM. As depicted in Fig. 3a, the three cell types, each represented in a different color, were visualized. The presence of the apical layer composed of a monolayer of alveolar cells (green) with incorporated MDMs (red), while at the basal side MDDCs (orange) were observed.

No noticeable change was observed in the cell morphology after exposure to different concentrations of single and aggregated AuNPs in comparison to control cultures exposed to NaCl_aq_ solution only (Fig. 3b-d).

The epithelial integrity of the cell layer after exposure was also assessed by testing the FITC-dextran (70 kDa) permeability. As shown in Fig. 3e, FITC-dextran translocation across the barrier 24 h after exposure to NaCl_aq_ solution was very low (1.95%) and remained low after 48 h (1.80%). Similar translocation rates were found for cells exposed to the highest concentration of single and aggregated AuNPs. In contrast, when the cells were treated with EDTA, a chelator agent known to open the tight junctions, dextran translocation increased to about 50%.

### Cell response after AuNPs exposure

**Fig. 4:**
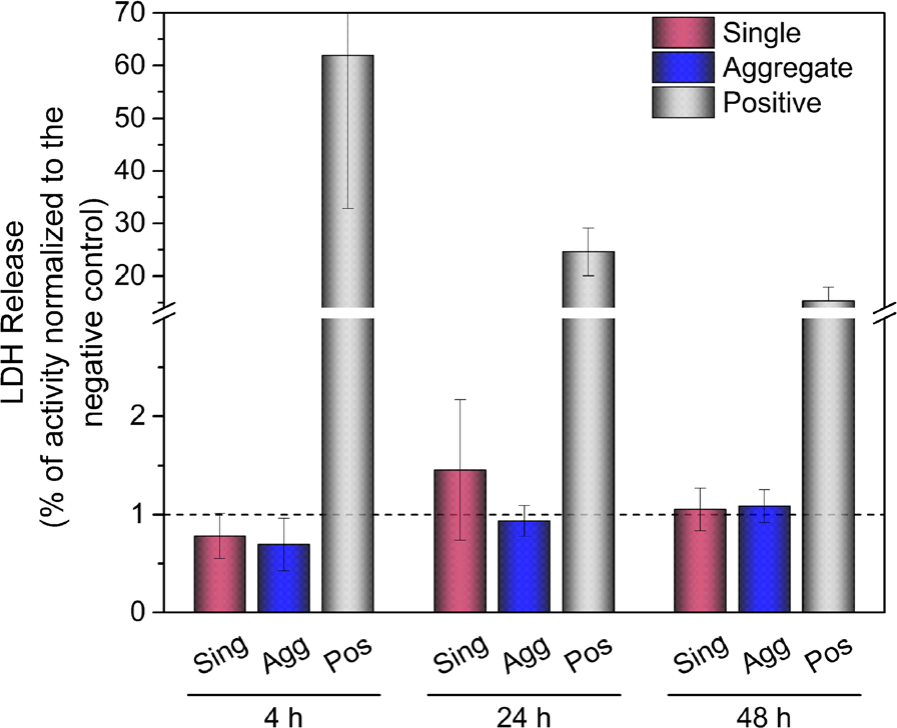
Cell viability estimated by quantification of the released lactate dehydrogenase enzyme after exposure to single and aggregated AuNPs at an applied dose of 300 ng/cm^2^. Values expressed as a mean (*n* = 3) after normalization to the negative control. Negative control: exposure to saline solution; Positive control: treatment with 0.1% triton X-100. Sing: single AuNPs; Agg: aggregated AuNPs

Post-exposure cytotoxicity was assessed by measuring the release of lactate dehydrogenase enzyme into the medium. As shown in Fig. 4, cell exposure to single and aggregated AuNPs up to the highest dose of 300 ng/cm^2^, did not show any apparent membrane damage 4, 24 and 48 h in comparison to saline solution.

(Pro)-inflammation response to AuNPs exposure was assessed by measuring the released cytokine TNFα. No TNFα release has been measured in comparison to the positive control indicating that the particles did not induce any pro-inflammatory reactions (data not shown).

### Biodistribution behavior of AuNPs and localization in the cells

**Fig. 5:**
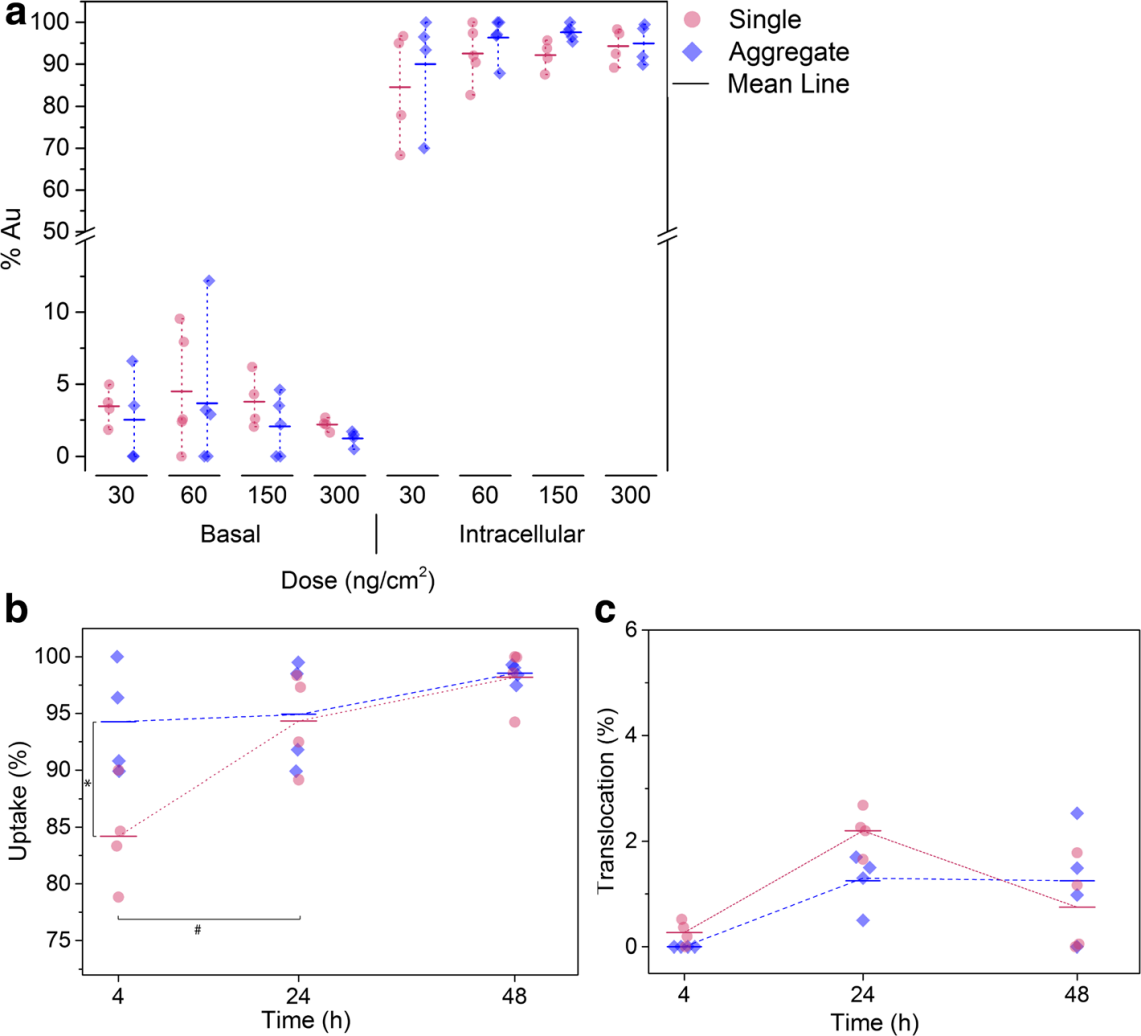
**a** Bio-distribution of single and aggregated AuNPs 24 h post-exposure. **b** Uptake kinetics after exposure to single and aggregated AuNPs at a concentration of 300 ng/cm^2^; significant differences of intracellular gold were found between single and aggregated AuNPs at 4 h (*) and for aggregated AuNPs after 4 and 24 h post-exposure (#), *p* < 0.05 was considered statistically significant. **c** Translocation kinetics after exposure to single and aggregated AuNPs at a concentration of 300 ng/cm^2^

After exposure onto the lung cell surface, AuNPs can either remain on the apical surface, being taken up by cells and/or translocated across the cell layer into the basolateral compartment. The AuNPs concentration was determined by ICP-OES for three different compartments: (i) air-exposed (apical) cell side to measure the AuNPs deposited on the apical cell surface, (ii) the intracellular AuNPs content, and (iii) the medium in the lower (basal) compartment to determine the translocated particles. The cultures were first exposed to single and aggregated AuNPs at different concentrations, i.e. 30, 60, 150 and 300 ng/cm^2^, and the gold distribution was analyzed 24 h after exposure (Fig. 5a). For both particle types and all concentrations less than 5% gold was found in the apical fraction and the majority of gold, i.e. more than 90% of the total applied gold mass, was found in the intracellular fraction (Additional file 1: Figure S7 shows amount of gold found intracellularly). Only exposure to single AuNPs at the lowest concentration of 30 ng/cm^2^ was slightly different as 12% of gold was found in the apical fraction and 85% intracellularly. However, for all conditions only a minor fraction of gold was found in the basolateral compartment showing minor translocation rates between 2 and 5%. Thus, the majority of single or aggregated AuNPs were taken up by the cells. Moreover, the translocation rate did not increase with increasing concentration.

Then, the biodistribution, i.e. uptake and translocation across the barrier, of exposed single and aggregated AuNPs was assessed at different time points, i.e. 4, 24 and 48 h at a dose of 300 ng/cm^2^ (Fig. 5b and c). For single AuNPs the cellular fraction after 4 h was 84%, increased to 94% after 24 h, and remained constant afterwards. For aggregated AuNPs 94% of the deposited mass was detected intracellularly after 4 h and remained constant. The results showed that the aggregated AuNPs were taken up faster than single AuNPs (94 vs 84%, respectively, at 4 h post-exposure), however no difference could be observed for the translocation rate.

**Fig. 6:**
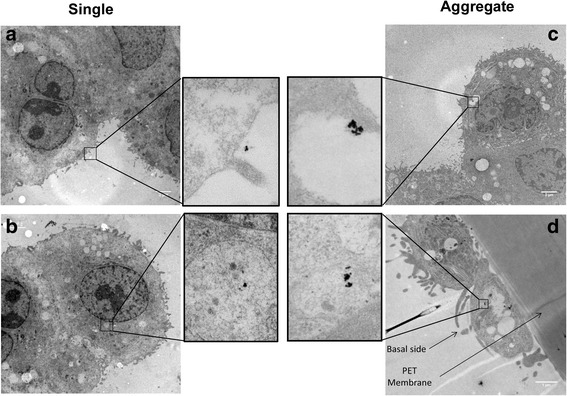
Cellular localization of single and aggregated AuNPs. **a** Single AuNPs attached to the outer apical cell surface. **b** Intracellular single AuNPs within vesicle on the apical side of the membrane. **c** Intracellular aggregated AuNPs found within vesicle on the apical side of the membrane. **d** Intracellular aggregated AuNPs found within vesicle in dendritic cell on the basal side. All the other black spots are not AuNPs and might originate from lead citrate staining (Histogram analysis in Additional file 1: Figure S8)

Ultrathin sections of cells exposed to AuNPs were visualized using TEM to observe the cellular localization of AuNPs (Fig. 6). After 24 h the majority of single and aggregated AuNPs were found intracellularly. Some of the particles were found to be attached to the outer apical cell surface and no particles were observed in the intercellular space. The AuNPs were found in all three cell types, i.e. MDM and epithelial cells on the upper insert surface, and even to a minor extent in MDDC on the basal side of the insert. Most of the AuNPs were localized in vesicles and only rarely in the cytoplasm. Attached or internalized aggregated AuNPs resulted in spot containing much higher density of AuNPs which is in line with the deposition characterization.

## Discussion

Lung is the first portal of entry into our body to airborne particles, which have been associated to lung and cardiovascular diseases [33, 34]. Aggregated NPs are a major form of airborne particles [7, 12]. Their low effective density compared to single particles of similar size increases their mobility and allow them to penetrate and deposit in the deep lung region [8]. However their behavior at the lung barrier is poorly studied, therefore gaining a better understanding of the aggregates interaction and fate at the human alveolar epithelial tissue barrier is important. In this study, an approach combining air liquid interface and advanced lung cell co-culture has been used representing a more realistic perspective when compared to submerged exposures [35]. Although the system has its limitation, i.e. it is not possible to follow the long-term fate of the particles and/or drugs in the blood as well as lymph circulations and secondary organs, it has been shown to give comparable results to in vivo data for short-term translocation kinetics, i.e. up to 24 h, of apically applied nanoparticles [36] or drugs [37].

AuNPs were used as model particles to study the interaction and biodistribution of single and aggregated NPs with lung cells after aerosol deposition. The well-controlled chemistry to synthesize AuNPs allowed the preparation of well-defined and stable aggregates composed of primary AuNPs with a core diameter of 14.5 nm, and a hydrodynamic diameter of 106 nm. These aggregated AuNPs were compared with their corresponding single AuNPs with a hydrodynamic diameter of 32 nm. Moreover, the scattering and absorption properties due to the LSPR are important aspects for their characterization and, together with possibility of gold traces quantification, they allow for exact characterizations including quantification [38].

Deposition of single and aggregated AuNPs after aerosolization using the air liquid interface cell exposure Cloud system was thoroughly characterized using TEM and ICP-OES techniques expressing deposition concentration in mass, number of entities and recovered surface area per surface area. Dose-controlled and reproducibility of the deposition were confirmed by the two techniques. At the highest concentration of 150 and 300 ng/cm^2^, aggregated NPs greatly reduced the number of deposited AuNP entities of 10 to 30 fold, respectively, which may have an impact on further cellular response. Furthermore, these analyses allowed to appreciate the deposition at a cellular level, as epithelial cells (A549) have a diameter of about 14 μm as reported by Jiang et al. [39], giving a total cellular surface of around 153 μm^2^, there is evidence to assume that even at lowest dose exposure of 30 ng/cm^2^, each cell should theoretically be in contact with 66 particles (Fig. 2b, applied dose: 0.05 mg/mL, deposition: 30 ng/cm^2^, 0.43 particles/μm^2^).

Exposure to single and aggregated AuNPs did not induce any significant adverse cellular effect regarding cytotoxicity, epithelial cell layer integrity and (pro-)inflammation. These results are in agreement with other studies who showed the biocompatible properties of AuNPs in vitro or in vivo [40]. The deposited mass of gold was in the range of 150 and 300 ng/cm^2^ which is higher as what is reported in animal studies, e.g. 0.2 to 8 ng/cm^2^ [22] or 3.3 ng/cm^2^ [41]. However, since there is no clear answer about the physiological relevance, i.e. occupation or biomedical concentration of gold nanoparticles via inhalation, and the aim was to compare the translocation rate of single AuNPs vs. aggregates the conditions producing reproducible deposition values without inducing any cytotoxicity were chosen.

The biodistribution of the AuNPs in the lung cells 24 h after exposure showed that majority of both single and aggregated particles were taken up and retained inside the cells, only a minor fraction translocated across the epithelial tissue layers, i.e. between 1.3 and 4.5%. The translocated fraction observed in this study is in agreement with another in vitro study where aerosolized 18-nm citrate AuNPs were exposed to a A549 epithelial cell monolayer where a translocation rate of 2% had been reported [36]. In vivo studies also reported only minor translocation of AuNPs across lung in rats, 0.5% after instillation of sulfonated triphenylphosphine AuNPs (18 and 200 nm) [22] and 1.4% after inhalation of citrate AuNPs agglomerates (peak diameter 45 nm) [25].

ICP-OES measurements showed that the uptake and/or translocation are fast processes as majority of, if not all, AuNPs were taken up and translocated already 4 h after exposure. This is in agreement with in vivo studies of inhaled AuNPs: one study in mice concluded that AuNPs (21 nm) were translocated after a short time, inferior to 2 h [41] and another study in rats investigating translocation over time showed that translocation of AuNPs (18–80 nm) was complete after 1 h [22].

The rapid translocation and the similar low rate (1.3–4.5%) regardless of the different deposited concentrations let suggest that translocation of single and aggregated AuNPs occurs through an active transcellular transport (or transcytosis) [21]. This hypothesis is further supported with TEM observations: 1) no AuNPs were found in the intercellular space; 2) most of the intracellular AuNPs were found in vesicles; 3) presence of AuNPs in MDDCs on the basal side of the membrane. Similar observations were found in stereological analysis of mice lung tissue after exposure to 21-nm AuNPs [42].

Although, aggregated AuNPs behavior was similar to single AuNPs regarding distribution, the only difference found was that aggregated AuNPs were faster observed intracellularly in comparison to single AuNPs (Fig. 5b). Indeed, it is well known that particle size and shape influence cellular uptake [21, 43]. However, this observation can be surprising since preferential uptake is commonly expected with particles of the size around 50 nm and with spherical shape [21]. The faster uptake of aggregated AuNPs could be explained by i) the larger surface area of the aggregated AuNPs; ii) a different cellular uptake pathway; iii) the lower number of deposited particles for aggregates. Firstly, several findings support the theory that larger surface area in contact with cell membrane allow for more multivalent ionic interactions explaining a faster or higher NPs uptake. For instance, a study investigating internalization of NPs with various sizes and shapes, found that in cylindrical NPs of similar volume, particles with higher aspect ratio were internalized faster, suggesting a favored internalization for the larger surface area [44]. Similar observations were found in a study comparing cellular uptake of single and aggregated transferrin coated AuNPs (30 and 98 nm, respectively) in which aggregated AuNPs uptake were 2-fold higher in comparison to single AuNPs in cells expressing few transferrin receptors [45]. Secondly, the difference observed in uptake kinetics could also be explained by a different uptake mechanism. Indeed, Kreyling et al. have observed a different translocation behavior with the bigger 200 nm AuNPs in comparison to 18–80 nm AuNPs, suggesting that these two NPs categories were endocytosed and/or exocytosed via different pathway [22]. Furthermore, a study investigating shape effect of mesoporous silica NPs found that spherical NPs were preferentially internalized via clathrin-mediated pathway while higher aspect ratio NPs favored caveolae-mediated pathways [46]. Finally, as shown in Fig. 2f, at a deposition of 300 ng/cm^2^, the number of deposited aggregated NPs is 30 times less than for single AuNPs.

## Conclusions

In this study, the biodistribution of aerosolized single and aggregated AuNPs was investigated using a 3D model of the human epithelial tissue barrier. Robust characterization was used to evaluate the exact delivered dose onto the cell surface and to determine the cellular uptake and translocation across the barrier. Overall, we found that within a short time (<4 h), the majority of the AuNPs, single or aggregated, were taken up and retained inside the cells while only a minor fraction translocated to the basal side (<5%). The low translocation rate is similar to the ones found for AuNPs in vivo highlighting the possibility of using a sophisticated in vitro approach to predict in vivo biokinetics of inhaled AuNPs. Finally, at higher concentration (300 ng/cm^2^) the aggregated AuNPs showed a significant reduction of the number of deposited spots and a faster cellular uptake but only during the first time points assessed, however, no significant change of the translocation rate was observed. Hence, aggregation is fundamental for the cellular uptake kinetics of NPs during the first hours after exposure and has to be considered, either in a biomedical setting of drug delivery or for hazard assessment.

## Additional file

Additional file 1: Supplementary information. (PDF 765 kb)
