# Supplementary material for: Biodistribution of single and aggregated gold nanoparticles exposed to the human lung epithelial tissue barrier at the air-liquid interface
Source: Part Fibre Toxicol. 2017 Nov 29;14:49. doi: 10.1186/s12989-017-0231-3 (PMC5707895; doi:10.1186/s12989-017-0231-3)
Supplement: Additional file 1: — Supplementary information. (PDF 765 kb) [file 12989_2017_231_MOESM1_ESM.pdf]

Supplementary Information

Biodistribution of single and aggregated gold nanoparticles exposed to the human epithelial tissue barrier at the air-liquid interface

AUTHOR NAMES

*Estelle Durantie[1], Dimitri Vanhecke[1], Laura Rodriguez-Lorenzo[1], Flavien Delhaes[1], Sandor Balog[1], Dedy Septiadi[1], Joel Bourquin[1], Alke Petri-Fink[1,2], Barbara Rothen-Rutishauser[1]**

1. Processing and Analysis of the TEM images

1.1. Single AuNPs

Single AuNPs images processing steps was performed as depicted in Fig. S1. Analyze particles measurements gave the number of particles, the diameter (major) and the area.

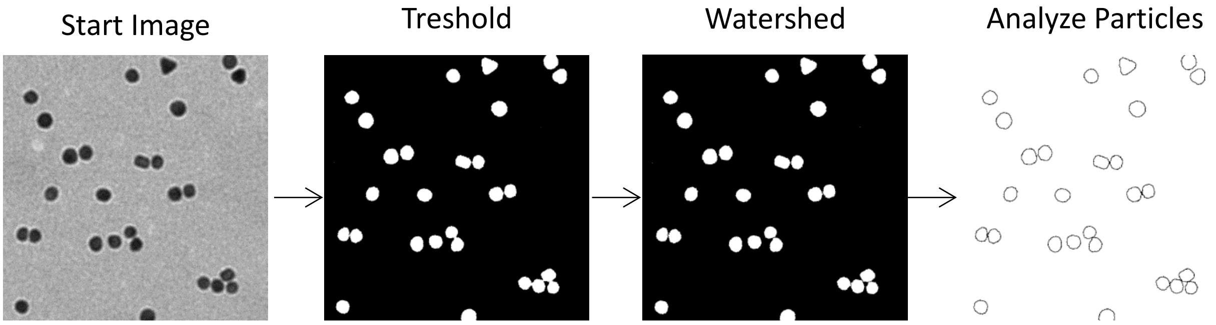

Fig. S1 Process analysis of single AuNPs images

1.2. Aggregated AuNPs

1.2.1. Number of aggregated AuNPs

To count the number of aggregated AuNPs, images processing steps was performed as depicted in Fig. S2.

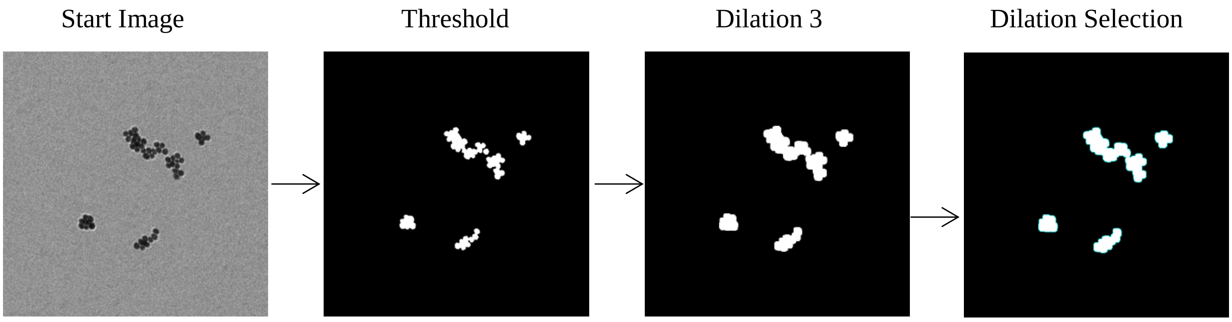

Fig. S2 Process analysis of aggregated AuNPs images to count the number of aggregated AuNPs

1.2.2. Area of aggregated AuNPs

To determine the surface area of aggregated AuNPs, images processing steps was performed as depicted in Fig. S3 starting from the dilation selection showed in Fig. S2.

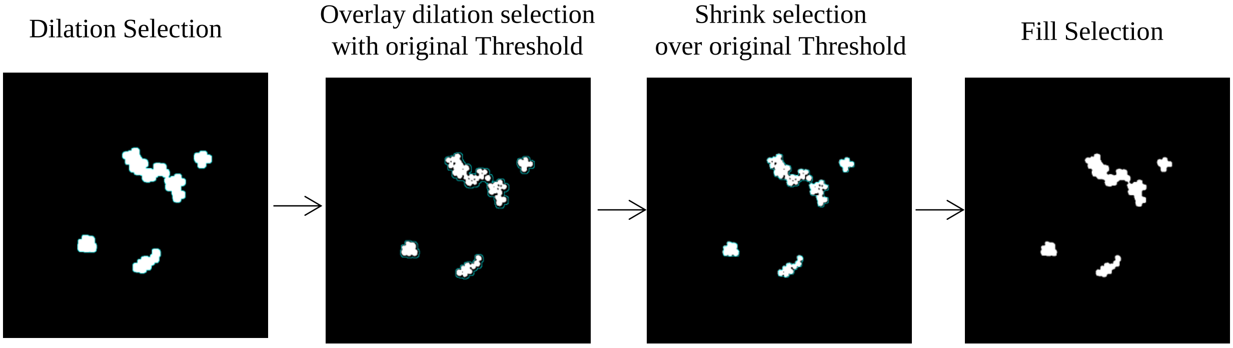

Fig. S3 Process analysis of aggregated AuNPs images to determine the area of aggregated AuNPs

1.2.3. Number of single AuNPs particle per aggregate

Transmission electron microscopy provides sufficient resolution to count single AuNPs, but delivers only 2D projections and no 3D data. This leads to possible bias when assessing particle numbers from micrographs, since particles within the aggregates may overlap.

If no particles are overlapping then the surface area of the projected shadow of an aggregate (A) is a function of the number of particles in the aggregate (N) and their radius ($\bar{r}$). Since the single particle analysis gave fairly monodispersed nanoparticles (average radius, 8.4 nm), the radius can be declared as a constant.

$$N = \frac{A}{\pi \cdot \bar{r}^2} \qquad \text{Equation (1)}$$

If particles are overlapping in the projection, then the estimated number of particles will be lower than the actual number of particles in the aggregate.

In order to estimate this bias (ξ), a sample of 48 randomly chosen aggregates was reconstructed in three dimensions by electron tomography and counted their exact particle numbers. Since the 3rd dimension was reconstructed, overlapping particles could be detected. In parallel we estimated the number of particles based on the projection area of each aggregate. These calculations were run on the sample obtained after aerosolization of the aggregated AuNPs at the highest concentration of 0.50 mg/mL, as it is the sample showing the most overlapping and so the higher risk of biased results.

1.3. Number of single and aggregated AuNPs

Table S1 Number of particles (N) measured for characterization of the deposition

**Mass deposited (ng/cm^2)**	**N Single**	**N Agg**
**30**	1550	270
**60**	4448	562
**150**	17385	722*
**300**	54869	1804

* Analysis was done on 2 single slot grids from 2 different experiments

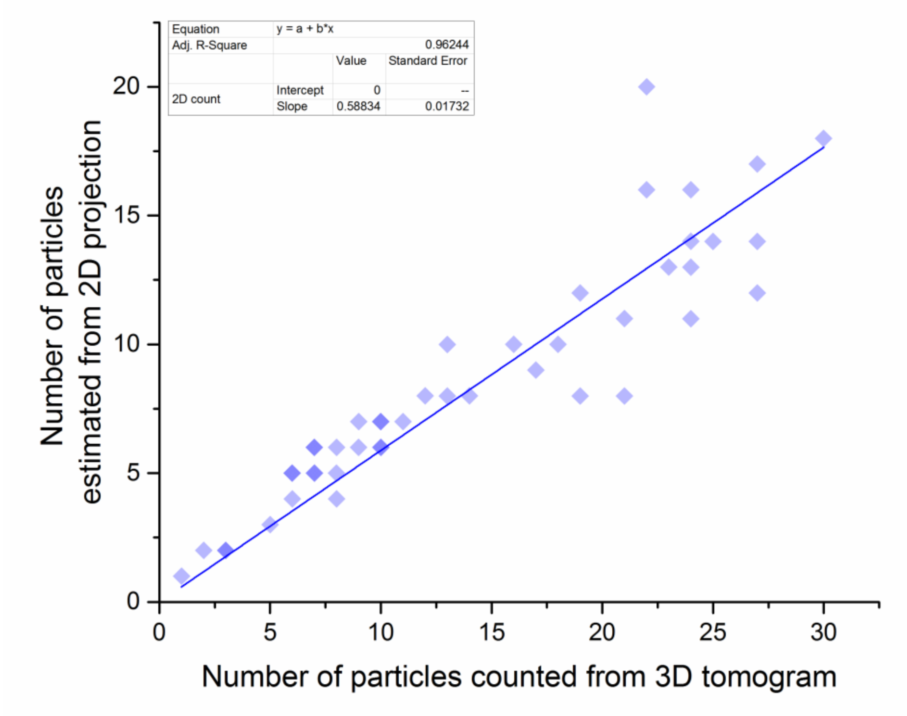

Fig. S4 Number of particles per aggregate, estimation of the bias using a 2D projection.

The results show that the actual number of particles and the estimated number of particles from 2D projections correlate in a linear way. Regression analysis (least squares) yield a slope of about 0.59, i.e. the number of particles estimated from the projected area is an underestimation of about 41%. A hetereoscedastic effect makes the prediction at larger aggregates increasingly imprecise.

$$\widehat{N} = \frac{1}{\xi} \cdot \frac{A}{\pi \cdot \bar{r}^2} \qquad \text{Equation (2)}$$

When correcting the number of particles estimated from 2D micrographs with this factor, no significant difference could be found between the actually counted and estimated number of particles.

2. Characterization of single and aggregated AuNPs suspension

2.1. TEM

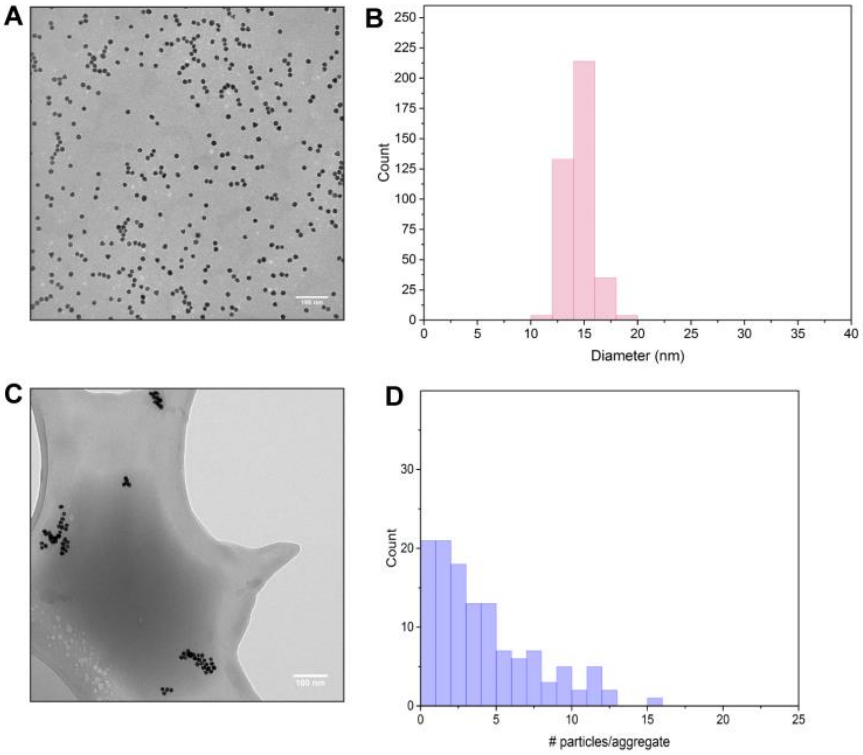

Fig. S5 TEM characterization of single and aggregated AuNPs in suspension: **A.** TEM image of single AuNPs. **B.** Size distribution of single AuNPs expressed as diameter (nm) (Major), N=390. **C.** cryo-TEM image of aggregated AuNPs. **D.** Size distribution of aggregated AuNPs expressed in number of particles per aggregate, N=110.

2.2. Zeta Potential

Table S2 Zeta Potential of single and aggregated AuNPs

	Single AuNPs	Aggregated AuNPs
**Zeta Potential (mV)**	+ 3.6	+ 29.0

Note: Even if zeta potential is different in the two systems, the number of amines in the polymer coating of the single and aggregated AuNPs was equal. The difference in zeta potential could be explained by a difference in the polymer conformation due to the difference of pH during AuNPs polymer coating.

3. Aerosolized aggregated AuNPs

The number of particles per aggregates was determined by application of the equation (2) to the surface area of the aggregate as detailed above.

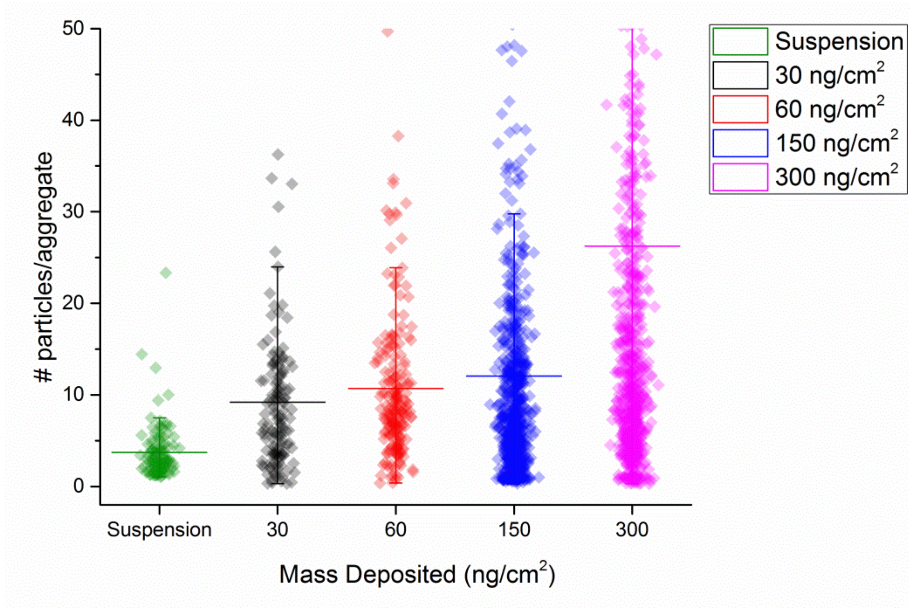

Fig. S6 Number of particles per aggregates in the aggregated AuNPs suspension (cryo-TEM) or after nebulization at the different concentration. Horizontal line represents the mean value, whisker represents the range within 1.5 interquartile range.

4. Intracellular amount of gold

The intracellular amount of gold 24 h post-exposure to single and aggregated AuNPs at different doses was determined by ICP-OES.

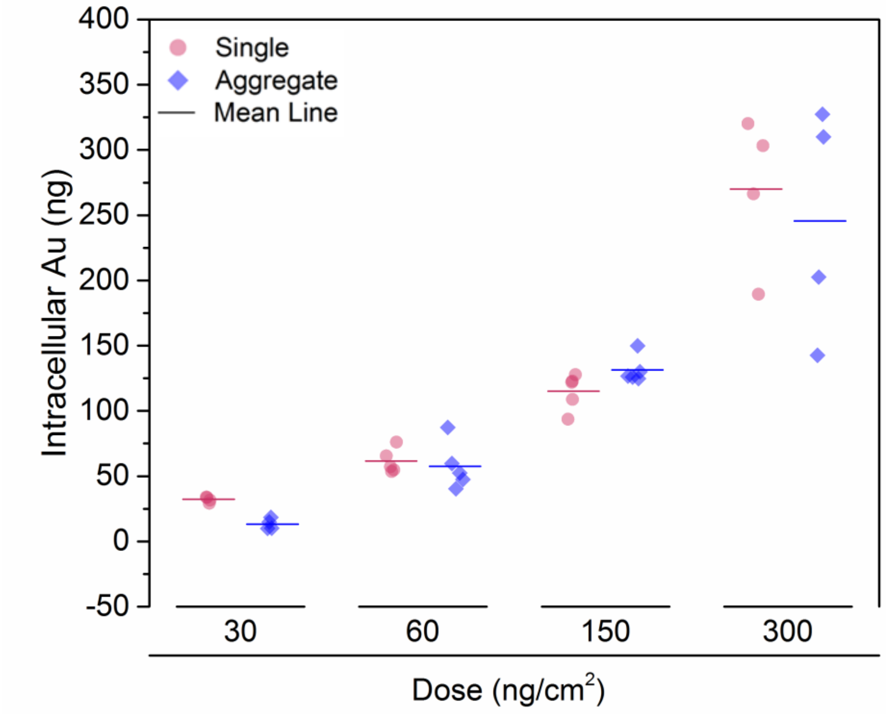

Fig. S7 Intracellular amount of gold 24 h post-exposure to single and aggregated AuNPs.

5. Cellular localization of AuNPs

To avoid any misinterpretation in the TEM images of cell cultures exposed to single and aggregated AuNPs in Fig. 6, only areas zoomed in contain AuNPs. Other dark spots in the images are not AuNPs and might originate from lead citrate staining. A histogram analysis of Fig. 6d is presented.

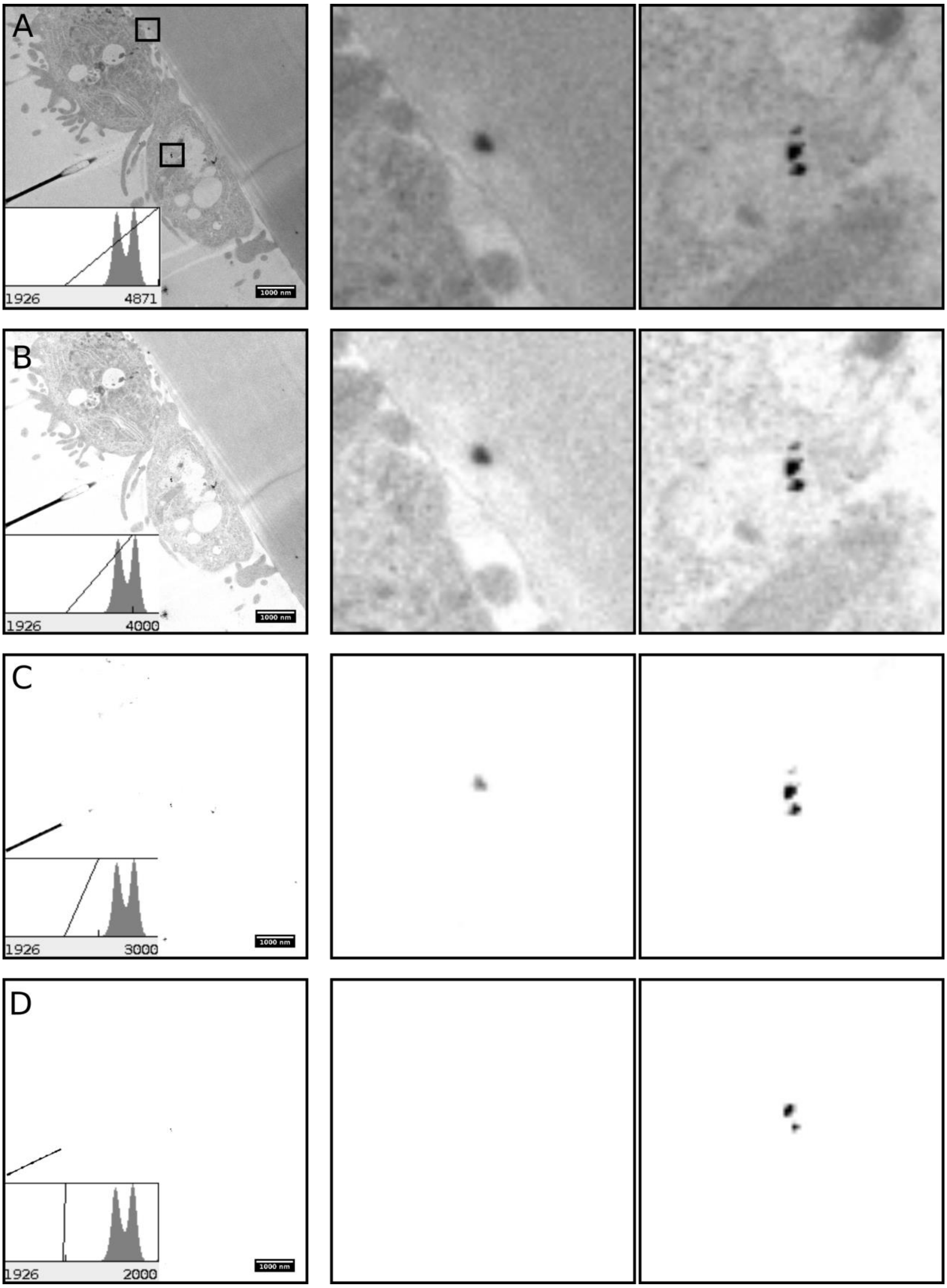

Fig. S8 Histogram analysis
